# Frey’s syndrome - unusually long delayed clinical onset post-parotidectomy: a case report

**DOI:** 10.4314/pamj.v5i1.56198

**Published:** 2010-04-07

**Authors:** Inchien Chamisa

**Affiliations:** 1 Kalafong Hospital, Department of General Surgery, University of Pretoria, South Africa

**Keywords:** Frey’s syndrome-parotidectomy-gustatory sweating

## Abstract

Frey’s syndrome is a complication of parotidectomy that is thought to occur as a result of aberrant regeneration of the postganglionic parasympathetic nerve fibres supplying the parotid gland to severed postganglionic sympathetic fibres which innervate the sweat glands of the face. Frey’s syndrome is difficult to treat but is a preventable phenomenon and surgeons must be aware of the available preventative methods during the initial surgery. An unusual case is presented involving a patient with delayed onset of Frey’s syndrome 40 years after parotidectomy in childhood. The potential for this long-delayed clinical presentation should be discussed with the patient before surgery in the parotid gland. Diagnostic methods, preventive measures and management options are briefly discussed.

## Background

Frey’s syndrome consists of gustatory discomfort, sweating and flushing of the skin overlying the parotid area which may be associated with pain in the auriculotemporal nerve distribution. It is caused by the severed ends of parasympathetic secretomotor fibres which innervated the salivary gland growing into the sweat glands of the skin.

Frey’s syndrome can be socially debilitating and because of the difficulty in its management, preventive measures should be instituted during the initial surgery. To our knowledge, the longest latency of Frey’s syndrome after parotidectomy recorded in the literature is 50 years [1]. Our patient had parotidectomy at the age of 7 years and presented 40 years later with Frey’s syndrome.

## Patient and case report

**Figure 1:**
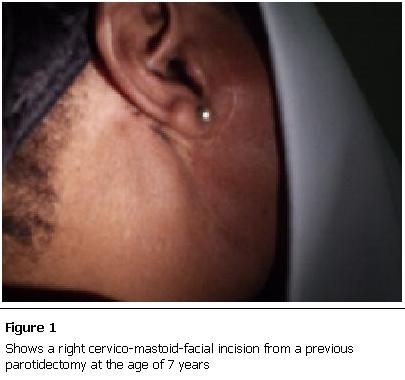
Shows a right cervico-mastoid-facial incision from a previous parotidectomy at the age of 7 years

A previously well 47 year old housewife presented to the surgical clinic with a 1 year history of worsening right-sided facial gustatory sweating and flushing associated with headaches and dizziness. She explained that the gustatory sweating was now socially embarrassing and she was desperate for a solution. At the age of 7 years she had undergone a parotidectomy for a parotid mass. There was nothing in the history to suggest why she had presented now rather than earlier. Physical examination confirmed a right cervico-mastoid-facial incision from the previous parotidectomy (Figure 1).

**Figure 2:**
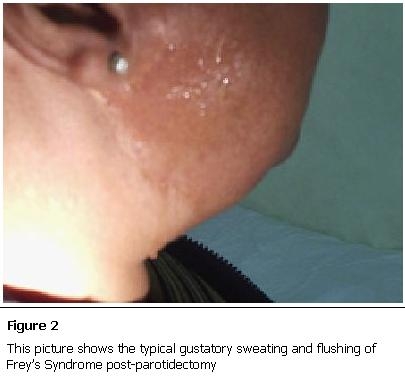
This picture shows the typical gustatory sweating and flushing of Frey’s Syndrome post-parotidectomy

She helpfully offered to show the signs as she munched on an apple and the gustatory sweating and flushing where immediately apparent as shown in figure 2. She was subsequently referred to the ear nose and throat (ENT) clinic for definitive management.

## Discussion

Frey’s syndrome is a disorder characterised by unilateral sweating and flushing of the facial skin in the area of the parotid gland occurring during meals. This syndrome was first described by Lucia Frey, a French neurologist in 1923. This condition is a sequela of parotidectomy and may follow other surgical, traumatic and inflammatory conditions of the parotid and submandibular glands. The presumed pathophysiology process is the aberrant regeneration of cut parasympathetic fibres between the otic ganglion and the salivary gland tissue leading to innervation of sweat glands and subcutaneous vessels. Gustatory stimulation then results in sweating and redness of the skin of the involved area [2].

The reported incidence of Frey’s syndrome after parotidectomy varies considerably depending on the method of assessment. Gustatory sweating is detected in almost 100% of cases, evaluated by means of a post-operative iodine-starch test (Minor test), but only 10-15% have serious complications [3]. The debilitating symptoms in Frey’s syndrome can be avoided with good preoperative planning and assessment. Thick skin flap and partial superficial parotidectomy are the most important techniques to minimize the risk of developing symptomatic Frey syndrome. An alternative is use of the superficial musculoaponeurotic system (SMAS) flap which is placed in the bed of the resected parotid gland. This serves as a protective barrier guarding against the aberrant anastomotic communications between the postganglionic secretomotor fibres and the adjacent sweat glands [4]. The ideal Frey’s syndrome barrier has to either remain in place permanently or be replaced by dense body fibrosis which prevents the growth of parasympathetic parotid fibres toward the facial skin sweat glands. In this regard, e-polytetraflouroethylene (PTFE) implants represent the ideal solution because of their good biocompatibility, low tissue reactivity and their lack of resorption. The incidence of Frey’s syndrome is also related to skin flap thickness in parotidectomy, with thin flaps developing significant symptoms. Thus Frey’s syndrome is a preventable phenomenon and the potential for its appearance should be discussed with the patient before surgery in the parotid gland.

Various methods have been developed to diagnose Frey’s syndrome, including the Minor’s starch-iodine test, thermography and use of questionnaires for the subjective assessment of symptoms. The Minor’s starch-iodine test is highly accurate and will identify asymptomatic patients with Frey’s syndrome. Thermography is a non-invasive test that provides a qualitative visual analysis of the cutaneous capillary response in Frey’s syndrome following parotid surgery.

Various forms of treatment of Frey syndrome, both medical and surgical, have been tried with varying degrees of success. However, the majority of patients are satisfied by an explanation of the condition and reassurance [5]. Intracutaneous injection of botulinum toxin is a safe and effective treatment with long-lasting effects for patients with extensive gustatory sweating [5]. Its use in Frey’s syndrome was initiated by Drobik and Laskawi in 1995. The neurotoxin enters the cytoplasm of nerve cells by endocytosis and neurotransmission is blocked until re-innervation occurs by collateral growth of fibres. Severe symptoms may justify tympanotomy and division of Jacobson’s nerve on the promontory of the medial wall of the middle ear.

## Conclusion

This case report serves to provide additional evidence of the possibility of a long-delayed clinical presentation of Frey’s syndrome post-parotidectomy. Frey’s syndrome can be socially debilitating and because of the difficulty in its management, preventive measures should be instituted during the initial surgery. Furthermore, patients should be warned of the possibility of this long-delayed clinical presentation.

## Patient consent

Written informed consent was obtained from the patient for publication of this case report and a copy of it is available for review by the Editor-in-Chief of this journal.

## Competing interests

No competing interests are involved in this case report
